# GDF8 Contributes to Liver Fibrogenesis and Concomitant Skeletal Muscle Wasting

**DOI:** 10.3390/biomedicines11071909

**Published:** 2023-07-06

**Authors:** Alexander Culver, Matthew Hamang, Yan Wang, Huaizhou Jiang, Jennifer Yanum, Emily White, Samer Gawrieh, Raj K. Vuppalanchi, Naga P. Chalasani, Guoli Dai, Benjamin C. Yaden

**Affiliations:** 1 Department of Biology, School of Science, Center for Developmental and Regenerative Biology, Indiana University-Purdue University Indianapolis, Indianapolis, IN 46202, USA; aeculver@indiana.edu (A.C.); hamang_matthew_joseph@lilly.com (M.H.); yan.wang3@bms.com (Y.W.); jyanum@iu.edu (J.Y.); 2 Department of Biological Sciences, College of Science, Purdue University, West Lafayette, IN 46202, USA; white491@purdue.edu; 3 Division of Gastroenterology and Hepatology, School of Medicine, Indiana University, Indianapolis, IN 46202, USA; sgawrieh@iu.edu (S.G.); rvuppala@iu.edu (R.K.V.); nchalasa@iu.edu (N.P.C.)

**Keywords:** liver injury, muscle atrophy, TGFβ family, Gdf8, liver–muscle crosstalk

## Abstract

Patients with end-stage liver disease exhibit progressive skeletal muscle atrophy, highlighting a negative crosstalk between the injured liver and muscle. Our study was to determine whether TGFβ ligands function as the mediators. Acute or chronic liver injury was induced by a single or repeated administration of carbon tetrachloride. Skeletal muscle injury and repair was induced by intramuscular injection of cardiotoxin. Activin type IIB receptor (ActRIIB) ligands and growth differentiation factor 8 (Gdf8) were neutralized with ActRIIB-Fc fusion protein and a Gdf8-specific antibody, respectively. We found that acute hepatic injury induced rapid and adverse responses in muscle, which was blunted by neutralizing ActRIIB ligands. Chronic liver injury caused muscle atrophy and repair defects, which were prevented or reversed by inactivating ActRIIB ligands. Furthermore, we found that pericentral hepatocytes produce excessive Gdf8 in injured mouse liver and cirrhotic human liver. Specific inactivation of Gdf8 prevented liver injury-induced muscle atrophy, similar to neutralization of ActRIIB ligands. Inhibition of Gdf8 also reversed muscle atrophy in a treatment paradigm following chronic liver injury. Direct injection of exogenous Gdf8 protein into muscle along with acute focal muscle injury recapitulated similar dysregulated muscle regeneration as that observed with liver injury. The results indicate that injured liver negatively communicate with the muscle largely via Gdf8. Unexpectedly, inactivation of Gdf8 simultaneously ameliorated liver fibrosis in mice following chronic liver injury. In vitro, Gdf8 induced human hepatic stellate (LX-2) cells to form a septa-like structure and stimulated expression of profibrotic factors. Our findings identified Gdf8 as a novel hepatomyokine contributing to injured liver–muscle negative crosstalk along with liver injury progression.

## 1. Introduction

The liver and skeletal muscle systems have a well-established interdependent endocrinological connection in both physiological and pathological states. One example of this inter-dependency is observed through their mutual contribution to the maintenance of glucose homeostasis through storage and metabolic mechanisms. In contrast, patients with liver cirrhosis exhibit abnormal capacity to store glucose as glycogen in skeletal muscle [1]. Moreover, skeletal muscle wasting, or sarcopenia, is recognized as a major complication in patients with cirrhosis, non-alcoholic fatty liver disease (NAFLD), and the more severe form known as nonalcoholic steatohepatitis (NASH) [2,3]. Recent clinical studies demonstrate a linear association of sarcopenia with severity of liver fibrosis in NASH patients [4,5,6]. In addition, the degree of muscle wasting is a strong correlate to adverse clinical outcomes and follow-up hospital costs in these patients [4,7,8,9,10,11,12]. Patients with cirrhotic livers and concomitant muscle atrophy tend to have lower survival rates and even more post-liver transplant complications. Paradoxically, most complications in liver cirrhosis patients resolve following a successful transplant, except for the underlying muscle wasting [13]. Thus, the ability to treat muscle mass loss is paramount for the clinical management of these patients [14,15,16]. Unfortunately, effective therapies are lacking because the signaling mediators of the liver–muscle axis remain unclear [17]. Therefore, it is a priority to identify and delineate the systemic cues that initiate and promote this sequela of liver diseases. 

Carbon tetrachloride (CCl_4_) is the most commonly used toxin to induce experimental liver fibrosis [18]. CCl_4_ is metabolized mainly by Cyp2E1 in hepatocytes. This process produces toxic free radicals causing massive centrilobular necrosis in the liver [19]. This in turn triggers inflammatory responses manifested by the activation of immune cells and the production of proinflammatory factors by these cells [20]. Subsequently, hepatocyte death and inflammation induce robust repair responses, indicated by hepatocyte proliferation and the activation of hepatic stellate cells (HSCs). Activated HSCs produce excessive collagens, initiating liver fibrosis [21]. The CCl_4_ model recapitulates human liver fibrosis in many aspects, including hepatocyte necrosis, inflammation, HSC activation, and liver fibrogenesis [18].

The TGFβ superfamily of secreted proteins include activins, growth differentiation factors (Gdfs), and bone morphogenetic proteins (BMPs). They engage with activin type II and type I receptor complexes to initiate Smad signaling to modulate organ development, growth, homeostasis, and repair [17,22,23]. Notably, it has been proposed that members of the TGFβ superfamily play integral roles in homeostasis and disease states of both the liver and skeletal muscle, and certain members may even serve as a nexus between the two organs [24,25,26]. In the present study, we set out to determine whether TGFβ ligands are causal mediators responsible for the deleterious communication between the injured liver with normal or injured skeletal muscle. We also evaluated the potential to prevent or reverse liver injury-induced muscle atrophy by inhibiting TGFβ superfamily signaling. We utilized both a fusion protein (ActRIIB-Fc) and a neutralizing antibody to myostatin (Gdf8) to demonstrate the importance of this signaling axis in this pre-clinical setting. ActRIIB-Fc is a fusion protein consisting of the extracellular ligand-binding domains of activin type IIB receptor with the Fc portion of mouse immunoglobulin G (IgG). ActRIIB-Fc primarily binds to and inhibits activins, Gdf8, and other TGFβ superfamily members [27]. We found that Gdf8 largely mediates the negative crosstalk of injured liver with skeletal muscle and simultaneously promotes liver injury. 

## 2. Materials and Methods

### 2.1. Approval of Animal Studies

All mouse experiments were performed with the approval of the Institutional Animal Care and Use Committee of Indiana University—Purdue University Indianapolis (SC337R) and are in accordance with the National Institute of Health Guide for the Care and Use of Laboratory Animals. For all studies described here, ten-week-old C57BL/6 female or male mice were used (Harlan, Indianapolis, IN, USA). Animals were housed in a room with controlled temperature (22 ± 2 °C) and a 12:12 h light–dark cycle (lights on at 6:00 a.m.) with ad lib access to food (TD 5001 with 0.95% calcium and 0.67% phosphorus, Teklad, Madison, WI, USA) and water.

### 2.2. Carbon Tetrachloride Liver Fibrosis Model

Hepatic fibrosis was induced by 10 mL/kg intraperitoneal injections of 1:10 diluted carbon tetrachloride (CCl_4_) (Sigma Aldrich, St. Louis, MO, USA) in corn oil twice weekly for a total of 6 to 9 weeks for chronic studies [28]. For acute studies, tissues were harvested 6, 24, or 48 h following a single injection of CCl_4_.

### 2.3. Skeletal Muscle Injury Model

Muscle injury was induced as previously described [29], with slight modifications. Briefly, muscle injury was induced by a 100 µL injection of a 10 µM cardiotoxin (CTX) (Sigma-Aldrich, St. Louis, MO, USA; part #C3987) solution into the gastrocnemius muscle with a three-point injection technique to fully cover the lateral and medial gastrocnemius. 

### 2.4. Gene Expression Analysis

Total RNA was extracted from isolated tissues using TRIzol reagent (Life Technologies, Grand Island, NY, USA) and treated with RNase-free DNase I (ThermoFisher, Waltham, MA, USA). RNA concentrations and A260/A280 ratios were determined by a NanoDrop Microvolume Spectrophotometer (ThermoFisher). RNA integrity was confirmed using RNA gel electrophoresis. Total RNAs (1 µg/sample) were reverse transcribed using a High-Capacity cDNA Archive Kit (Applied Biosystems, Foster City, CA, USA). The cDNAs were assayed for genes of interest using TaqMan Gene Expression Analysis (Applied Biosystems) and quantified by the 2^−ΔΔCt^ method. The initial hold step (50 °C for 2 min followed by 95 °C for 10 min) and 40 cycles of a two-step PCR (92 °C for 15 s and 60 °C for 1 min) were performed.

### 2.5. Protein Quantification

Tissue lysates were generated using 1 mL per 100 mg tissue in lysis buffer (Cell Signaling Technologies, Danvers, MA, USA). Total protein contents were quantified using a Pierce BCA Protein Assay Kit (Thermo Scientific). Gdf8 and Smad2/3 proteins were quantified by ELISA methods (GDF8/Myostatin Quantikine ELISA kit, Cat #DGDF80, R&D Systems, Minneapolis, MN, USA; PathScan phospho-Smad2 Ser465/467/Smad3 ser423/425 and total for Smad 2 and 3, kits #12001, 12002, 7244, Cell Signaling, Danvers, MA, USA). Procedures were followed according to the manufacturer’s guidelines. 

### 2.6. Histology and Immunohistochemistry 

Muscle or liver tissue was evaluated using hematoxylin and eosin (H&E) or Masson’s trichrome staining (Abcam, Cambridge, MA, USA). For each muscle, distribution of the fiber diameter was calculated by analyzing ~200 myofibers using digital slide scanning (ScanScope XT, Aperio, Vista, CA, USA). A collagen proportionate area (CPA) was quantified using the HALO Image Analysis Platform (Albuquerque, NM, USA) of liver sections stained with Masson’s trichrome. FFPE liver sections were assayed for Gdf8 content via a commercial antibody (LSBio, LS-C37420/170820, Washington, DC, USA). 

### 2.7. Human Liver Sample Collection

Liver tissue was collected from individuals with established cirrhosis awaiting liver transplantation at the time of their liver transplantation procedure in the operating room. Demographic data, etiology of cirrhosis, and other relevant information such as medication, alcohol use, and smoking history were captured at the time of enrollment. Liver tissue samples were snap frozen using liquid nitrogen and stored at −80 °C until use. All samples were collected and handled equally except for the duration of storage. This study was reviewed and approved by the Institutional Review Board (IUPUI IRB: EX0904-11).

### 2.8. ActRIIA-Fc and ActRIIB-Fc Proteins and Antibodies

The ActRIIA-Fc and ActRIIB-Fc proteins described in this report were expressed in stably transfected Chinese hamster ovary cells and were generated at Eli Lilly (Indianapolis, IN, USA). Isolation of the chimeric proteins from concentrated cell culture supernatants was performed using a two-step purification method. In the first step, crude conditioned cell culture media containing the specific variant was captured onto Mab select sepharose (GE Healthcare, Buckinghamshire, UK) under high salt conditions (1 M sodium chloride) and eluted using a step-gradient of 10 mM sodium citrate, pH 3.0. Pooled protein was concentrated using an Amicon Ultra-15 concentrator (Millipore, Boston, MA, USA) and further purified using a Superdex G200 preparative gel-filtration step (GE Healthcare, Buckinghamshire, UK). These steps generally resulted in protein purity of > 95%, as assessed by SimplyBlue staining (Invitrogen, Carlsbad, CA, USA) SDS-PAGE and analytical gel filtration on a TSKG3000SWXL column (Tosoh Bioscience, Tokyo, Japan). GDF8 antibody was generated by Eli Lilly and Company and its properties were described previously [30,31]. Activin A antibody was purchased from R&D System (Cat #MAB3381, Minneapolis, MN, USA). 

### 2.9. Body Composition

Lean body mass and fat mass were measured in live conscious animals using nuclear magnetic resonance (NMR; Echo Medical Systems, Houston, TX, USA). 

### 2.10. Cell Culture and In Vitro Assays

Experiments were performed using primary hepatocytes isolated from mice. Briefly, under anesthesia, the peritoneal cavity was opened, and the liver was perfused in situ via the portal vein for 4 min at 37 °C with calcium–magnesium (CM)-free HEPES buffer and for 7 min with CM-free HEPES buffer containing type IV collagenase (35 mg/100 mL) and CaCl_2_ (10 mM). Cells were used only if the cell viability was above 90% as assessed by trypan blue exclusion. After three centrifugations (44 g for 2 min) in Leibovitz’s L-15 washing media supplemented with 0.2% bovine albumin, cells were plated onto 24-well or 96-well plates (26,000 cells/cm^2^). Cells were cultured in high-glucose (25 mM) DMEM supplemented with 10% FBS. All culture media contained penicillin (100 units/mL) and streptomycin (100 μg/mL). After cell attachment for 2 h, the medium was replaced with fresh medium supplemented with 10% fetal bovine serum (FBS). PMH cultures were maintained under 5% CO_2_ atmosphere at 37 °C. CCl_4_ or corn oil was administered at 0.5% volume directly onto plated hepatocytes for 24 h prior to media collection. C_2_C_12_ myoblasts were obtained from ATCC (Manassas, VA, USA) and were thawed and plated per ATCC protocols in 10% FBS-supplemented media. Prior to cells reaching confluence, they were differentiated in growth media supplemented with 2% horse serum for 5 days with daily media changes. Collected hepatocyte media were diluted 1:10 into myoblast differentiation media and supplemented with IgG or ActRIIB-Fc at 100 ng/mL prior to addition to C_2_C_12_ cells, whereas corn oil media received no treatment. After 5 days of daily media change, muscle cells were imaged and myotube length quantified using Aperio ImageScope 12.3 software. Hepatocyte media aspartate aminotransferase (AST) and alanine aminotransferase (ALT) were assayed with a Hitachi instrument. LX-2 cells, a human hepatic stellate cell line, was a gift from Dr. Scott L. Friedman from the Mount Sinai School of Medicine (New York, NY, USA). They were cultured in DMEM supplemented with 2% FBS (Gibco, Invitrogen, Carlsbad, CA, USA).

### 2.11. Data Analysis and Presentation

All results were expressed as mean ± standard error of the mean (SEM). All datasets were assessed for normality via Shapiro–Wilk test. Statistical analysis was performed by ordinary ANOVA if normality assessments were passed or by Kruskal–Wallis if normality assessments were not passed. Significant differences were defined when *p*-value ≤ 0.05. 

## 3. Results

### 3.1. Acute Liver Injury Induces Rapid and Negative Responses in Skeletal Muscle

**Figure 1:**
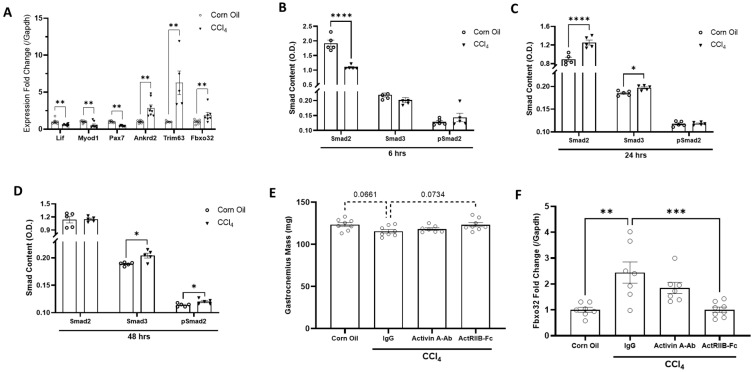
**CCl_4_-induced liver injury acutely activates muscle catabolic gene markers and downregulates satellite cell markers.** Female mice were administered a single dose of corn oil or carbon tetrachloride (CCl_4_). Muscle samples were collected at 6, 24, and 48 h post CCl_4_ injection. (**A**) Muscle mRNA expression of the genes indicated was analyzed 6 h after livery injury. (**B**–**D**) Protein levels of muscle total Smad 2 and Smad 3 and phosphorylated Smad 2 in muscle were measured via ELISA 6, 24, and 48 h post injury. In a subsequent study, female mice received IgG, activin A-Ab, or ActRIIB-Fc (10 mg/kg) 16 h prior to a single administration of CCl_4_. (**E**) Gastrocnemius muscle wet weight and real-time PCR analysis of (**F**) muscle Fbxo32 78 h following CCl_4_ injection are shown. Data are expressed as mean ± SEM (*n* = 5 mice/group). Significance is indicated as * *p* ≤ 0.05, ** *p* ≤ 0.01, *** *p* ≤ 0.001, or **** *p* ≤ 0.0001. *p*-values between 0.05 and 0.1 are indicated by dotted lines.

To understand how liver injury affects skeletal muscle, we evaluated the acute muscle response to a model of liver injury induced by carbon tetrachloride (CCl_4_) [32]. This model consistently demonstrates muscle wasting reminiscent of clinical observations of liver disease patients [33]. As early as 6 h following a single CCl_4_ administration, the expression of ubiquitin ligase Trim63 and Fbxo32, critical regulators of early muscle turnover [34], was markedly increased and persisted for 3 days (Figure 1A). Additionally, expression of three transcription factors (Lif, Myod1, and Pax7) essentially required for myogenesis were rapidly downregulated, whereas ankyrin repeat domain 2 (Ankrd2), a powerful regulator of myogenesis and stress responses, was upregulated (Figure 1A). Smad proteins are intracellular signal-transducing proteins known to be regulated by TGFβ family members to modulate myogenesis. We observed alterations in total Smad 2 or 3 protein content 6–24 h post CCl_4_ injection and increases in phosphorylated Smad2 2 days after CCl_4_ exposure (Figure 1B–D). These data indicate that skeletal muscle sensitively and negatively responds to liver injury at least via a mechanism that suppresses satellite cell markers, potentially limiting the muscle’s regenerative capacity and potential long-term preservation of lean mass. Moreover, the observed increases in muscle Smad2 activity following CCl_4_ dosing strongly support a role for TGFβ signaling in the muscle’s response to acute liver injury. 

### 3.2. Neutralization of ActRIIB Ligands but Not Activin A May Prevent the Initiation of Liver Injury-Induced Muscle Atrophy

To evaluate whether ActRIIB ligands mediate the initiation of injured liver–muscle communication in vivo, female mice were treated with IgG, activin A antibody (activin A-Ab), or ActRIIB-Fc 16 h prior to a single dose of CCl_4_. Activin A was included in this study because it has been suggested by others to be the most likely ActRIIB receptor ligand responsible for muscle mass loss [35]. Three days following CCl_4_ injection, acute liver injury reduced muscle mass, whereas ActRIIB-Fc but not activin A antibody prevented this event (Figure 1E). However, these changes did not reach statistical significance, which was most likely due to the short period of exposure of the muscle to the injured liver. Notably, the expression of ubiquitin ligase Fbxo32, a potent muscle atrophy promotor, was increased in acutely injured liver, which was attenuated by ActRIIB-Fc but not activin A-Ab (Figure 1F). These data suggest that ActRIIB-binding ligands modulate the initiation of muscle atrophy after liver injury and appear to be independent of activin A.

### 3.3. Neutralization of ActRIIB Ligands Prevents Chronic Liver Injury-Induced Muscle Atrophy Independent of Gender

**Figure 2:**
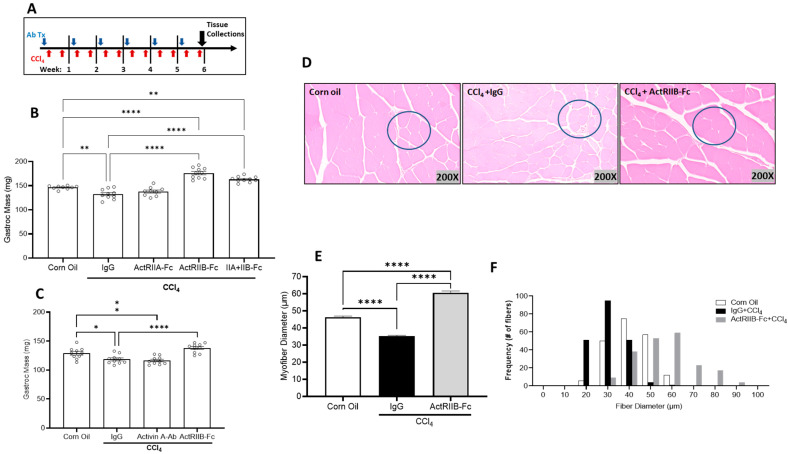
**CCl_4_-induced chronic liver injury induces muscle atrophy in mice independent of sex.** (**A**) Male mice were chronically administered carbon tetrachloride (CCl_4_) twice a week for 6 weeks. Sixteen hours before the 1st CCl_4_ injection each week, male mice received corn oil, immunoglobulin (IgG), ActRIIA-Fc, ActRIIB-Fc, or a combination of ActRIIA-Fc and ActRIIB-Fc (10 mg/kg). A corn oil (non-injured) group was added as a homeostasis control. After 6 weeks of CCl_4_ treatment, (**B**) gastrocnemius wet weights were evaluated. Female mice underwent the same CCl_4_ injury paradigm for 6 weeks while receiving IgG, Activin A-Ab, or ActRIIB-Fc weekly. (**C**) Gastrocnemius mass was assessed by wet weight. (**D**) Representative H&E cross-sectional images of myofibers in the gastrocnemius muscle. (**E**) Average fiber diameter and (**F**) the frequency distribution of gastrocnemius muscle fibers were analyzed. Data are expressed as mean ± SEM (*n* = 10). Significance is indicated as * *p* ≤ 0.05, ** *p* ≤ 0.01, or **** *p* ≤ 0.0001. All quantifications of myofibers (~200 myofibers counted per group) were determined using ImageScope 12.3 software (Aperio).

To elucidate whether ActRIIB ligands mediate progressive muscle mass loss caused by chronic liver injury, male mice were administered CCl_4_ twice weekly for 6 weeks. Sixteen hours before the first CCl_4_ injection each week, mice received weekly IgG, ActRIIA-Fc, ActRIIB-Fc, or a combination of these two fusion proteins, which function as pharmacological ligand traps for their respective receptors (Figure 2A). As a result, CCl_4_-induced chronic liver injury caused significant muscle mass loss, which was prevented by ActRIIB-Fc but not ActRIIA-Fc. Treatment with ActRIIB-Fc and ActRIIA-Fc combination did not show additive effects in muscle mass relative to ActRIIB-Fc alone, demonstrating that an activin receptor IIB-specific ligand is primarily responsible for the induction of muscle atrophy (Figure 2B). These data suggest that inactivation of ActRIIB ligands prevents muscle atrophy during chronic liver injury.

We performed a similar study to the one described above in female mice. Female mice received weekly IgG, activin A-Ab, or ActRIIB-Fc along with twice weekly CCl_4_ administrations (Figure 2A). Chronic liver injury induced by CCl_4_ resulted in a decrease in muscle mass, which was prohibited with ActRIIB-Fc but not activin A-Ab (Figure 2C). Further analysis showed that, in response to chronic liver damage, the muscle exhibited reduced myofiber diameter, whereas ActRIIB-Fc treatment fully prevented this event (Figure 2D–F). Collectively, our results indicate that chronically injured liver negatively communicate with skeletal muscle independent of gender, and TGFβ family ligands neutralized by ActRIIB-Fc appear to mediate this negative crosstalk.

### 3.4. Neutralization of ActRIIB Ligands Reverses Chronic Liver Injury-Induced Muscle Atrophy

**Figure 3:**
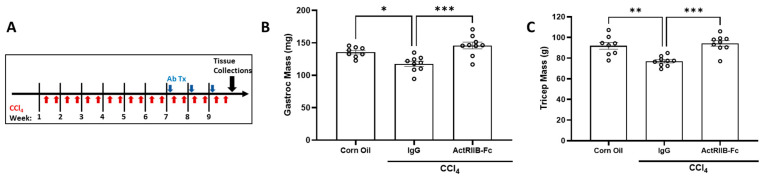
**Skeletal muscle mass loss induced by existing CCl4 injury was reversed with ActRIIB inhibition.** (**A**) Female mice were injected with CCl_4_ or corn oil twice per week for 6 weeks. Then, mice were dosed with ActRIIB-Fc or IgG once per week for 3 weeks, during which CCl_4_ or corn oil injections continued (9 weeks total). (**B**) Gastrocnemius and (**C**) triceps brachii muscle masses were assessed by wet weight at study completion. Data are expressed as mean ± SEM (*n* = 9). Significance is indicated as * *p* ≤ 0.05, ** *p* ≤ 0.01, or *** *p* ≤ 0.001.

To evaluate whether ActRIIB ligand inhibition can reverse atrophied muscle after chronic liver injury, we first dosed female mice twice weekly with CCl_4_ for six weeks. Following that, mice treated with either ActRIIB-Fc or IgG were dosed weekly for 3 additional weeks with continued CCl_4_ injections (Figure 3A). Consequently, chronic liver injury caused muscle mass loss, which was reversed by ActRIIB-Fc (Figure 3B,C). The results further demonstrate that ActRIIB ligands mediate the negative crosstalk of injured liver to muscle. 

### 3.5. Injured Liver Produces Gdf8 in Both Humans and Mice

Gdf8 is a myokine expressed in muscle that potently suppresses muscle mass and myogenesis in an autocrine/paracrine manner. It has high affinity to ActRIIB, and this receptor–ligand interaction inhibits proliferation and activation of satellite cells and myoblasts via downregulation of Pax7 and Myod1 expression in muscle [36,37,38,39]. Recent studies have hypothesized a role of Gdf8 in liver disease with concomitant sarcopenia, and it is associated with poor survival outcomes in cirrhosis patients [40,41]. Moreover, Gdf8 has been shown to regulate the fibrogenic phenotype of hepatic stellate cells in vitro, implying a role in hepatic injury response [42,43]. Thus, we hypothesized that Gdf8 may transduce negative signaling from injured liver to muscle. To test this, we performed the following studies.

**Figure 4:**
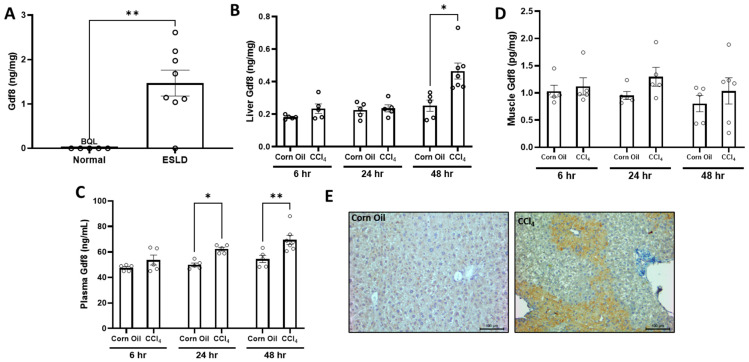
**Hepatic Gdf8 content is increased in human patients with end-stage liver diseases (ESLD) similar to acutely injured mouse liver.** (**A**) Liver samples were collected from healthy individuals (*n* = 5) or patients with established cirrhosis (*n* = 7). Liver lysates were prepared and subjected to quantification of Gdf8 protein via ELISA. All normal control samples were below quantifiable limits of the assay. (**B**–**E**) Male mice (*n* = 5) were administered a single dose of corn oil or carbon tetrachloride (CCl_4_). The concentration of Gdf8 protein was measured via ELISA in the (**B**) liver, (**C**) plasma, and (**D**) muscle at the time points indicated after CCl_4_ injection. Liver sections were generated from mice 48 h post single CCl_4_ injection and immune-stained for Gdf8. (**E**) Representative images of Gdf8 immunostaining are shown. Data are expressed as mean ± SEM. Significance is indicated as * *p* ≤ 0.05, ** *p* ≤ 0.01.

We first assayed human cirrhotic liver samples and observed abundant Gdf8 protein expression, whereas it was virtually undetectable in healthy liver (Figure 4A). In male mice, within 48 h after a single dose of CCl_4_, acutely damaged liver produced increased Gdf8 protein along with elevated circulating Gdf8 (Figure 4B,C). No significant changes in muscle Gdf8 protein were observed (Figure 4D). Immunohistochemistry detected abundant Gdf8 protein in pericentral (Zone 3) hepatocytes in injured liver (Figure 4E). This finding is consistent with the well-characterized function for zone 3 hepatocytes in processing xenobiotics and explains why fibrosis manifests pericentrally in the CCl_4_ model. This indicates that hepatocytes metabolizing CCl_4_ produce Gdf8 protein, representing a novel source of this TGFβ ligand in a pathological condition. 

**Figure 5:**
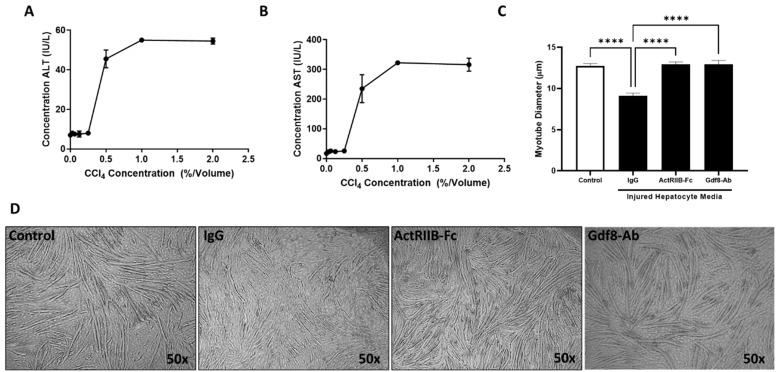
**Damaged primary hepatocytes release Gdf8, which directly acts on C_2_C_12_ cells.** Primary mouse hepatocytes were exposed to various concentrations of CCl_4_ as indicated for 24 h. (**A**) ALT and (**B**) AST in the culture medium were analyzed. Hepatocyte media were collected following exposure to 0.5% CCl_4_ by volume. The hepatocyte media were then diluted 1:10 into C2C12 differentiation media daily for 5 days along with IgG, ActRIIB-Fc, or Gdf8-Ab (100 ng/mL for each). (**C**) Myotube diameter was measured by ImageScope software (Aperio) after 5 days of myotube differentiation. (**D**) Representative images of differentiation myotubes. Data are expressed as mean ± SEM (*n* = 3 wells/group). Significance is indicated as **** *p* ≤ 0.0001.

To ascertain how muscle directly responds to liver injury, primary mouse hepatocytes were damaged via exposure to 0.5% CCl_4_ in culture medium. The concentration was determined as the minimal concentration to cause maximal release of liver enzymes (Figure 5A,B). ActRIIB-Fc soluble decoy receptor or Gdf8 antibody was added to differentiating C_2_C_12_ myotubes along with diluted injured hepatocyte or control medium with an equivalent CCl_4_ concentration. Culture medium from insulted hepatocytes but not medium from healthy hepatocytes inhibited myotube formation (myogenesis), as assessed by the reduced myotube diameter. The addition of an ActRIIB-Fc or a Gdf8 antibody fully rescued myotube formation potential (Figure 5C,D). This observation suggests that injured hepatocytes release ActRIIB-binding ligand(s), mainly Gdf8, which may exert direct adverse effects on muscle. 

### 3.6. Neutralization of Gdf8 Prevents Chronic Liver Injury and Concomitant Muscle Mass Loss

**Figure 6:**
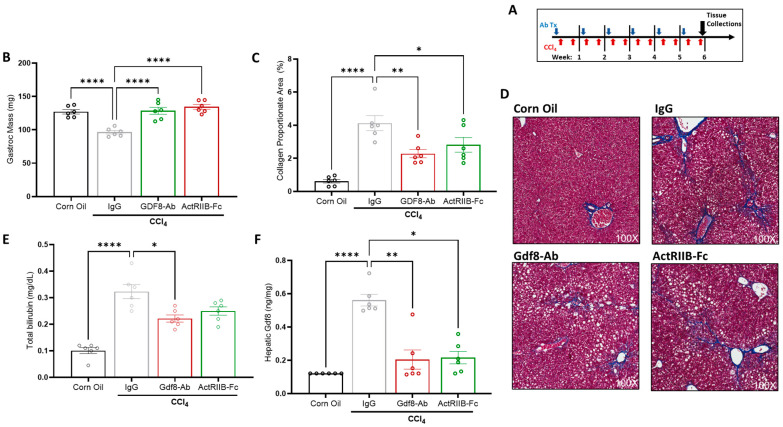
**Gdf8-Ab or ActRIIB-Fc prevents muscle atrophy and hepatic fibrogenesis in CCl_4_-induced chronic liver injury.** (**A**) Male C57BL/6 mice were chronically administered carbon tetrachloride (CCl_4_) twice a week for 6 weeks. Sixteen hours before the first CCl_4_ injection each week mice received IgG, Gdf8-Ab, or ActRIIB-Fc. A corn oil (non-injured) group was added as a homeostasis control. After 6 weeks of CCl_4_ injury, (**B**) gastrocnemius mass, (**C**,**D**) hepatic collagen proportionate area, and (**E**) total bilirubin levels were quantified and (**F**) hepatic Gdf8 levels were measured via ELISA. The minimally detectable values were used for the corn oil control group. (**D**) Representative images of Masson’s trichrome staining on liver sections. Data are expressed as mean ± SEM (*n* = 6). Significance is indicated as * *p* ≤ 0.05, ** *p* ≤ 0.01, or **** *p* ≤ 0.0001. Collagen proportionate area was quantified using HALO image analysis software.

Gdf8 appeared to mediate injured liver–muscle crosstalk, and thus Gdf8 neutralization may lead to the maintenance of muscle mass in the context of chronic liver injury. To test this, male mice were treated weekly with either IgG, Gdf8-Ab, or ActRIIB-Fc and administered CCl_4_ twice weekly for 6 weeks (Figure 6A). Consequently, we found that Gdf8 antibody and ActRIIB-Fc completely and equivalently protected against CCl_4_-induced skeletal muscle mass loss (Figure 6B). Surprisingly, we observed that Gdf8 neutralization also reduced hepatic collagen deposition (Figure 6C,D) and circulating bilirubin levels (Figure 6E) to a similar extent as ActRIIB-Fc treatment. Furthermore, hepatic Gdf8 protein content was increased with chronic CCl_4_ injury and reduced with Gdf8-Ab and ActRIIB-Fc treatment (Figure 6F). These results together demonstrate that Gdf8 neutralization inhibits liver fibrogenesis, improves liver function, and simultaneously prevents concomitant muscle atrophy. 

### 3.7. Neutralization of Gdf8 Reverses Chronic Liver Injury and Concomitant Muscle Mass Loss

**Figure 7:**
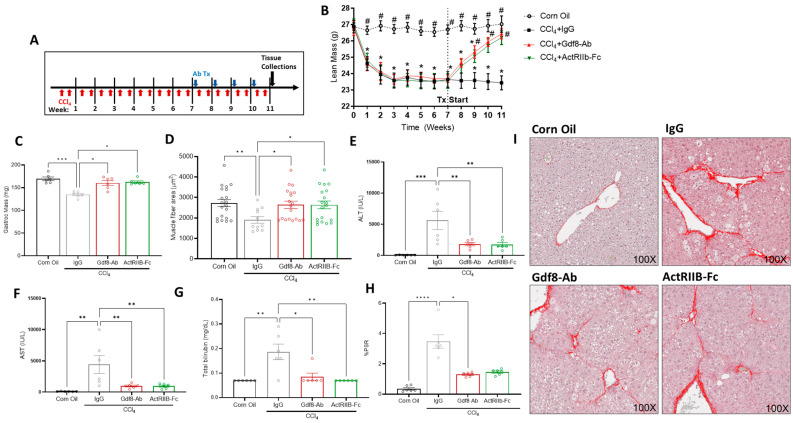
**Gdf8-Ab or ActRIIB-Fc reverses muscle atrophy and hepatic fibrogenesis in CCl_4_-induced chronic liver injury.** (**A**) Male C57Bl/6 mice were chronically administered CCl_4_ or corn oil twice a week for 11 weeks. Starting in week 7, 16 h before the first weekly CCl_4_ injection, mice received a weekly dose of IgG, Gdf8-Ab, or ActRIIB-Fc with (**B**) continual total body lean mass quantification via QNMR. After 4 weeks of CCl_4_ + antibody treatment, (**C**) gastrocnemius mass and (**D**) muscle fiber cross-sectional areas were assessed. Plasma (**E**) ALT, (**F**) AST, and (**G**) total bilirubin were measured in terminal blood. (**H**) Percent area of collagen stain was analyzed from images of Picrosirius red staining on liver sections. (**I**) Representative Picrosirius red staining on liver sections are shown. Data are expressed as mean ± SEM (*n* = 10). Significance is indicated as * *p* ≤ 0.05 compared to corn oil control and as # *p* ≤ 0.05 vs. IgG for LBM QNMR data. For all other figures, significance is indicated as * *p* ≤ 0.05, ** *p* ≤ 0.01, *** *p* ≤ 0.001, or **** *p* ≤ 0.0001. The collagen proportionate area was quantified using HALO image analysis software.

To assess the therapeutic potential of Gdf8 neutralization in an existing state of liver disease, we injured male mice twice weekly with CCl_4_ for a period of 11 weeks. Starting from the eighth week, mice received either anti-Gdf8 Ab, ActRIIB-Fc, or IgG once per week (Figure 7A). Compared to IgG controls, anti-Gdf8 therapy largely recovered the lost lean mass (Figure 7B), gastrocnemius mass (Figure 7C), and myofiber cross-sectional area (Figure 7D) observed in the CCl_4_-injured animals, similarly to ActRIIB-Fc treatment. Simultaneously, both ActRIIB-Fc and anti-Gdf8 antibody treatments significantly improved liver injury and function, manifested by reduced circulating liver enzymes ALT (Figure 7E) and AST (Figure 7F) and total bilirubin (Figure 6G). Concordantly, Gdf8 neutralization reduced hepatic collagen deposition as measured by picrosirius red staining, again, similarly to ActRIIB-Fc (Figure 7H,I). Collectively, these results demonstrate that hepatic Gdf8 is largely responsible for transducing adverse signaling effects of injured liver to skeletal muscle, and that Gdf8 promotes hepatic fibrotic response to chronic liver injury. 

**Figure 8:**
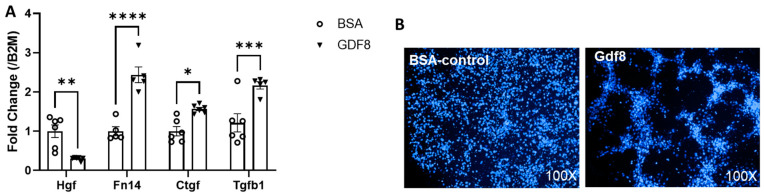
**Gdf8 activates human hepatic (LX-2) cells in vitro.** Human hepatic stellate cells (LX-2) were treated with bovine serum albumin (BSA) or Gdf8 protein at equal concentrations (100 ng/mL) for 24 h. (**A**) Real-time PCR analyses of Hgf, Fn14, Ctgf, and Tgfb1 mRNA expression were performed. Data are expressed as mean ± SEM (n = 6). Significance is indicated as * *p* ≤ 0.05, ** *p* ≤ 0.01, *** *p* ≤ 0.001, or **** *p* ≤ 0.0001. (**B**) Representative DAPI-stained images (100X) of LX-2 cells. B2M, β2-microglobulin.

To determine whether Gdf8 exerts a direct effect on hepatic stellate cells, the major contributor to liver fibrogenesis, we exposed human hepatic stellate (LX-2) cells to exogenous Gdf8 at a concentration of 100 ng/mL [42]. We found that Gdf8 induced a gene expression signature indicative of hepatic stellate cell activation manifested by decreased expression of Hgf and increased expression of Fn14, Ctgf, and Tgfβ1 (Figure 8A). Remarkably, Gdf8 protein stimulated morphological changes in LX-2 cells, redolent of a septa-like structure commonly observed in liver fibrosis (Figure 8B). These results suggest that increased Gdf8 following liver injury may directly act on hepatic stellate cells to promote liver fibrogenesis. 

### 3.8. Liver Injury Negatively Affects Muscle Regeneration, Which Is Prohibited by Neutralization of ActRIIB Ligands

**Figure 9:**
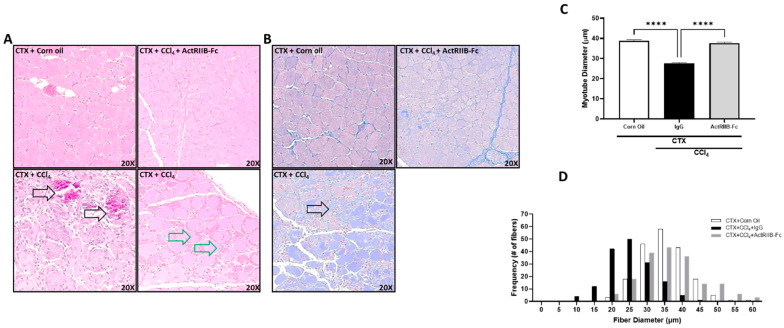
**CCl_4_-induced liver injury negatively affects muscle repair following cardiotoxin (CTX) injury.** Female mice were injected with carbon tetrachloride (CCl_4_) or corn oil every three days over a span of 10 days (3 administrations). Focal muscle injury was induced via direct CTX injection in the gastrocnemius muscle 6 h after receiving the first CCl_4_ treatment. ActRIIB-Fc or IgG was dosed prior to CTX injection. Muscle samples were harvested at day 10 post CTX injection. (**A**) Representative H&E cross-sectional images of myofibers in the gastrocnemius muscle. Black arrows indicate areas of calcification (bottom left) and green arrows indicate defective myocyte regeneration (bottom right). (**B**) Representative trichrome staining of muscle sections (20X). Arrow indicates fibrotic regions. (**C**) Fiber diameter of gastrocnemius muscles and (**D**) the frequency distribution of corresponding fibers (white—corn oil control, black—CCl_4_, and light gray—ActRIIB-Fc) were evaluated. All quantifications of myofibers (~200 counted per group) were determined using ImageScope 12.3 software (Aperio). Data are expressed as mean ± SEM (*n* = 6). Significance is indicated as **** *p* ≤ 0.0001.

Due to the finding that liver injury causes myogenic satellite cell marker suppression (Figure 1A), we queried whether liver injury would adversely affect muscle repair (myogenesis) and, if so, whether TGFβ family members also mediate this effect. To answer this question, we treated female mice with CCl_4_ or corn oil every three days for a period of 10 days. A single dose of cardiotoxin (CTX) was injected into the gastrocnemius muscle 6 h after the first CCl_4_ administration to induce focal muscle injury and repair [29]. IgG or ActRIIB-Fc was dosed prior to CTX treatment. As a result, 10 days after CTX injection, simultaneous liver and muscle injury led to skeletal muscle calcification (Figure 9A) and fibrosis (Figure 9B). We observed blunted regeneration in the removal of the necrotic sarcoplasm, followed by concurrent regeneration within the myotube membrane around the necrotic cytoplasm (Figure 9A). In addition, prominent collagen deposition and replacement of skeletal muscle tissue with non-muscle cells (Figure 9B) and significant reduction in size for nascent fibers (Figure 9C,D) were found with concomitant liver and muscle injury. ActRIIB-Fc treatment prevented these defects in muscle repair (Figure 9A–D). Taken together, this demonstrate that chronic liver injury results in detrimental effects on muscle repair after focal injury, strongly suggesting a role for hepatic dysfunction to inhibit muscle satellite cell activation and myogenesis. Furthermore, these effects were blunted by ActRIIB-Fc-mediated inhibition of TGFβ members, suggesting that extracellular ligand signaling through ActRIIB mediates the liver–muscle crosstalk in this context. 

### 3.9. Exogenous Gdf8 Disrupts Muscle Regeneration, Mimicking Liver Injury

**Figure 10:**
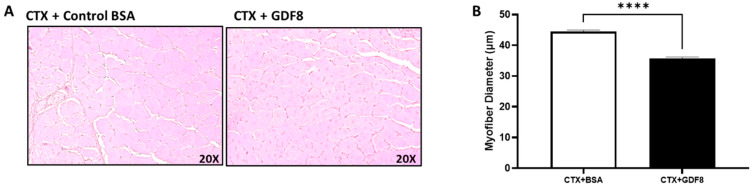
Exogenous Gdf8 disrupts muscle regeneration. In female mice, muscle injury was induced by CTX, and 2 h later, 5 µg of Gdf8 or bovine serum albumin (BSA) were directly injected into the muscle. The next day, 1 µg of each was injected to mimic the injury-mediated waning in ligand exposure. Muscle samples were collected at day 10 post CTX injury. (A) Representative H&E cross-sectional images of myofibers in the gastrocnemius muscle. (B) Fiber diameter of gastrocnemius muscles. All quantifications of myofibers (~200 counted per group) were determined using ImageScope 12.3 software (Aperio). Data are expressed as mean ± SEM (*n* = 5). Significance is indicated as **** *p* ≤ 0.0001.

Our in vitro study shows that, like ActRIIB-Fc, Gdf8 inhibiting antibody recovered the diameter of myotubes exposed to injured hepatocyte media (Figure 5C,D). This finding suggests that liver injury-derived changes in systemic Gdf8 could mediate the negative effect of injured liver on skeletal muscle repair. To demonstrate this, muscle injury was induced via CTX and, 2 h later, 5 µg of Gdf8 protein or BSA were injected directly into the muscle. The next day, 1 µg of Gdf8 protein was administered to the injury site. Doses were chosen to mimic the injury-mediated waning in ligand exposure, as the protein is quickly absorbed and degraded. Ten days post CTX injury, muscles treated with Gdf8 displayed a decrease in regenerating myofiber diameter compared to the BSA control group (Figure 10A,B), reflecting earlier observations with CCl_4_. This result strongly suggests that Gdf8 contributes to liver injury-induced disruption of myofiber regeneration. 

These studies demonstrate that ActRIIB-Fc intervention prevents and reverses liver injury-induced muscle atrophy and regenerative capabilities. We show that injured liver produces significant Gdf8 in both humans and mice. Furthermore, neutralization of Gdf8 largely recapitulates the positive muscle effects of ActRIIB-Fc intervention. Thus, these findings allow us to propose that ActRIIB ligands, primarily Gdf8, are significant mediators transducing the adverse signaling effects of injured liver to muscle. We and others have previously demonstrated that activin A induces muscle atrophy and degeneration [44]. However, in the context of CCl_4_-induced liver injury, we did not observe overt effects of neutralization of activin A alone on muscle atrophy. These finding suggests that activin A may not contribute to the injured liver–muscle crosstalk in our preclinical model. 

Previous studies by others have suggested several potential mediators connecting liver injury and muscle wasting, including hyperammonemia, insufficiency of growth hormone, and testosterone [40,45,46,47,48,49,50]. Liver dysfunction and portosystemic shunting causes impaired ureagenesis and thus hyperammonemia, a consistent metabolic complication in cirrhotic patients [51,52]. A NF-kB/myostatin pathway in muscle cells has been proposed to contribute to ammonium acetate-induced muscle degeneration [40]. In addition, growth hormone was shown to inhibit the expression of myostatin in skeletal muscle [53]. These findings propound Gdf8 as a signal mediator downstream of those potential mechanisms. Indeed, cirrhotic patients exhibit increased Gdf8 expression in both skeletal muscle and plasma [54,55]. Gdf8 is considered an autocrine/paracrine myokine and a potent suppressor of myogenesis [38,39]. Here, we demonstrate for the first time that liver injury/fibrosis drives the production of hepatic Gdf8 to negatively regulate skeletal muscle mass. We thereby propose Gdf8 as a candidate hepato-myokine during liver injury. The data prompt the question as to whether Gdf8/ActRIIB ligand signaling is a key central mechanism by which the liver communicates with muscle more broadly in hepatic injury and disease, including NASH and cirrhosis. 

One intriguing finding from our studies identified a circulatory environment that impedes muscle injury and repair. Our pathological assessment of the combination of liver and muscle injuries unveiled a discovery very redolent of a myopathy where the presence of Ringbinden fibers exists (Figure 9A). Ringbinden fibers have been described as an aberrant form of myofibrils that encircle themselves around existing or even dead fibers. Ringbinden fibers have been found predominantly in fast-twitch fibers in mice that possess a mutation in the skeletal muscle α-actin gene (Acta1) [56], which would be consistent in our model, wherein CCl_4_-induced liver injury selectively affects glycolytic fibers and is reinforced by Gdf8′s ability to affect glycolytic composition and type II fibers.

Previously published findings have demonstrated muscle dysfunction in CCl_4_-induced liver injury. Specifically, Weber et al. showed an increase in muscle protein catabolism of rat hindlimb muscles in a manner dependent on the severity of liver injury in CCl_4_-injured animals [57]. In that study, the muscle effects of CCl_4_ were limited to fast glycolytic fibers and did not reflect a more diffuse toxic effect in the muscle even in the context of 10-fold increases in CCl_4_ exposure on the muscle due to phenobarbital administration. Additionally, myotoxin-induced damage and degeneration can be characterized by specific histopathological features consistent with myofiber damage and immune cell infiltration [58], and this was not observed in our CCl_4_ studies or in other publications to our knowledge. These findings reported by Weber et al. are in line with more recent published data by Giusto et al. showing increased muscle protein degradation as the mechanism responsible for muscle atrophy observed in a CCl_4_-induced liver injury model [33]. Giusto et al. also reported increases in muscle protein degradation following several weeks of repeated CCl_4_ administration attributable to NF-KB-mediated upregulation of the ubiquitin ligase trim63, which is known to be regulated in muscle by myostatin [59]. Importantly, Giusto et al. also showed reductions in muscle myostatin (Gdf8) protein content following several weeks of CCl_4_-induced liver injury [33]. Although this may seem initially contradictory to reported clinical reports of increased circulating myostatin levels in liver disease patients who present with muscle atrophy [40,41], our data suggest that muscle may not be the sole source of increased circulating Gdf8 observed in these patients. Ultimately, although we cannot exclude the direct effects of CCl_4_ on damaging muscle, we do conclude that muscle Gdf8 expression is not significantly increased following hepatotoxic injury with CCl_4_, and the observed increased circulating levels are primarily attributable to extra-skeletal muscle sources, i.e., liver and potentially other organs. 

The mechanisms by which Gdf8 contributes to the pathogenesis of liver damage are not entirely clear at this time, but multiple studies have shown that Gdf8 functions as a profibrotic factor in various tissues [41,42,43,60,61,62]. Our data have corroborated this role in the liver by demonstrating the ability of Gdf8-Ab to reduce CCl_4_-induced liver fibrosis. We postulate that, in addition to the endocrine role in inducing muscle atrophy, Gdf8 may play an additional autocrine role by stimulating fibrosis. This was highlighted by the morphological and gene expression changes engendered by Gdf8 that are indicative of stellate cell activation in vitro, which revealed Gdf8 as a novel and pivotal mediator, which potentially originates from damaged hepatocytes, underlying the potential crosstalk between hepatocytes and HSCs in damaged liver. Other reports show that Gdf8 induces the expression of procollagen type 1, TGFβ1, and the tissue inhibitor of metalloproteinase-1 in LX-2 cells and stimulate the release of procollagen type I from these cells [42], in line with our findings. 

Our studies demonstrate inhibition of multiple members of the TGFβ superfamily as a promising therapeutic strategy to treat both primary- and secondary-organ injury or multi-organ injury. Here we demonstrate that targeting ActRIIB ligands, especially Gdf8, generates dual beneficial effects on both injured liver and wasted muscle. Our findings highlight that in scenarios where liver injury exists, it is vital to not only provide therapies that protect the liver and augment its repair but also address the resultant muscle atrophy that is a consequence of factors released into the systemic circulation shortly after the liver insult. Here we focused our efforts mainly on evaluating the effects of ActRIIB ligands on muscle atrophy secondary to liver injury. We also revealed that ActRIIB ligands may modulate liver fibrogenesis by directly targeting HSCs. 

## 4. Conclusions

**Figure 11:**
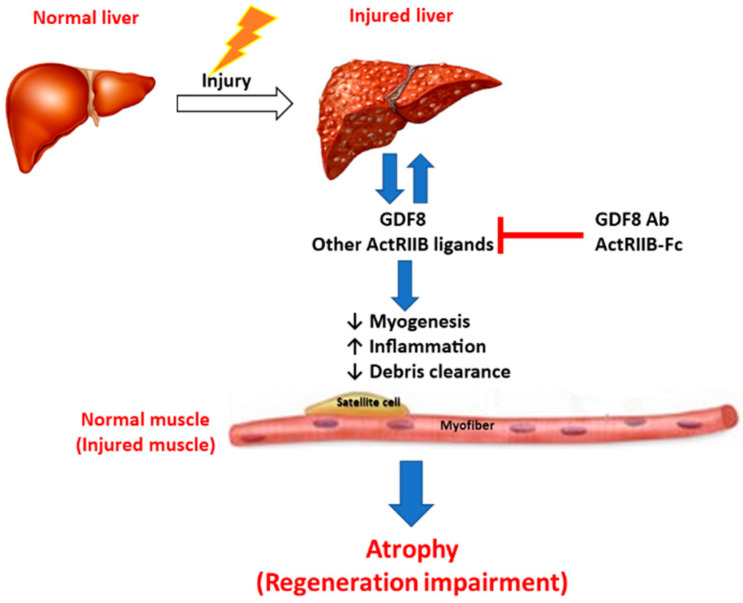
**Hypothesis of liver and skeletal muscle communication under pathological conditions.** Injured liver produces and releases Gdf8 and potentially other ActRIIB ligands, promoting liver injury progression and simultaneously causing systemic disruption of TGFβ signaling in skeletal muscle and likely other organs. ActRIIB ligands, mainly Gdf8, induce myofiber atrophy and inhibit myogenesis, as well as various biological processes, including inflammation and tissue remodeling, resulting in muscle degeneration and regeneration impairment. Degenerating skeletal muscle may in turn negatively feedback onto both the liver and skeletal muscle, promoting the progression of liver injury/fibrosis and concomitant muscle atrophy. Thus, simultaneous inhibition of ActRIIB ligands, especially Gdf8, is a powerful approach to preventing or reversing muscle atrophy concomitant to liver injury and even improving both injured liver and degenerating muscle.

Based on our findings, we identified a working hypothesis to provide a substrate for future investigations to define potential new therapies (Figure 11). Recently, cirrhotic patients and the coinciding muscle wasting have been well documented [4,5,6]. Here we revealed that livers in patients with ESLD also produce abundant Gdf8. Furthermore, we identified specific hepatic cell types in NASH patient liver biopsies that may drive increased Gdf8 expression in fibrotic tissue. It is worth noting that we cannot definitively confirm that increases in Gdf8 observed in those patients is solely attributed to hepatic expression. Indeed, the increases observed in our studies are still relatively low compared to basal Gdf8 expression in muscle, and there are likely additional tissues involved that warrant further investigation. Nonetheless, these findings demonstrate the future potential of therapeutics targeting TGFβ family members for the treatment and control of liver diseases associated with muscle atrophy. Indeed, clinical evidence already exists for this pathway regulating skeletal muscle via ActRIIB-Fc treatment in the form of ACE-031, which has been shown to increase total lean body mass and thigh muscle volume in patients with muscular dystrophy [16]. Furthermore, antibody therapy directed against ActRIIB (BYM338-Bimagrumab) also demonstrates the ability to increase lean body mass in the clinical setting [63]. Recent BYM388 data in type II diabetic and obese patients demonstrated efficacy to improve outcomes associated not only with glucose control but also with hepatic fat fraction reductions, a finding highly relevant to NAFLD and NASH pathologies [64]. Results from these clinical studies may provide information pertinent to the utility of such therapeutics in liver disease.

## Data Availability

The data presented in this study are available on request from the corresponding authors.
